# Does atorvastatin have augmentative effects with sodium valproate in prevention of migraine with aura attacks? A triple-blind controlled clinical trial

**DOI:** 10.1186/s40780-021-00198-8

**Published:** 2021-04-01

**Authors:** Reza Ganji, Nastaran Majdinasab, Saeed Hesam, Nazanin Rostami, Mehdi Sayyah, Adeleh Sahebnasagh

**Affiliations:** 1 Department of Clinical Pharmacy, Faculty of Pharmacy, Jundishapur University of Medical Sciences, Ahvaz, Iran; 2 Musculoskeletal Rehabilitation Research Center, Department of Neurology, Jundishapur University of Medical Sciences, Ahvaz, Iran; 3 Department of Biostatistics and Epidemiology, School of Health, Ahvaz Jundishapur University of Medical Sciences, Ahvaz, Iran; 4 Student Research Committee, Ahvaz Jundishapur University of Medical Sciences, Ahvaz, Iran; 5 Education Development Center, Ahvaz Jundishapur University of Medical Sciences, Ahvaz, Iran; 6 Clinical Research Center, Department of Internal Medicine, North Khorasan University of Medical Sciences, Bojnurd, Iran; 7 Department of Surgical Medicine, Faculty of Medicine, North Khorasan University of Medical Sciences, Imam Ali Hospital, Shahriar Street, Bojnourd, North Khorasan Province Iran

**Keywords:** Headache, Migraine with Aura, Sodium valproate, Atorvastatin, Prophylaxis

## Abstract

**Background:**

Migraine is a painful and disabling nervous disorder which negatively affects the quality of life. Migraineurs may suffer from a generalized vasomotor dysfunction. Statins improve vasomotor and vascular function, with their pleiotropic effects. We aimed to assess efficacy and safety of adding Atorvastatin to prophylactic regimen in better control of migraine with aura.

**Methods:**

This triple-blind controlled clinical trial was on 68 patients with migraine with aura. An interval of at least 1 month was given to evaluate vitamin D3 level and eligibility. In patients with vitamin D3 deficiency, the correction with vitamin D supplementation was provided. The patients were randomly assigned to receive atorvastatin 20 mg plus sodium valproate 500 mg or placebo plus sodium valproate 500 mg once a day for 2 months. The patients were evaluated based for the number of attacks and pain severity based on Visual Analogue Scale.

**Results:**

There was a significant (*p* = 0.0001) improvement in severity of pain and number of migraine attacks by adding Atorvastin to the prophylactic regimen of patients with migraine with aura. After controlling for variable parameters, the differences between two arms of the study was yet statistically significant (*p* = 0.0001). A significant number of participants in intervention group were satisfied by their treatment (*p* = 0.001) with no remarkable side effects (*P* = 0.315).

**Conclusions:**

Adding atorvastatin to migraine with aura preventive regimen may help reduce the number of acute attacks and pain severity without causing considerable side effects and led to a better patient satisfaction.

**Trial registration:**

IRCT20180106038242N1. Registered: 7 February 2018.

## Background

Headache is one of the most common medical complaints and disabling nervous disorders. More than 90% of people experience at least one headache attack per year [1]. Globally, 240 million people are estimated to suffer from 1.4 billion headache attacks, annually [2]. Hence, treatment of headache has been a medical priority [3]. Migraine is the most common type of chronic headache. According to the International Headache Society, migraine is characterized by recurrent, benign, and pulsating headache which involves one side of the head and may last for as long as 72 h [4]. Migraine may be caused by some certain known stimuli. Migraineurs suffer from nausea, vomiting and other symptoms of nervous dysfunction [5]. Approximately, 90% of patients have a positive family history, which place them at a higher risk for disease development. The severity and frequency of migraine attacks tend to diminish over time [6]. According to the World Health Organization, migraine was ranked as the 19th cause of disability worldwide [7]. Since only half of patients with headache seek medical care, it is difficult to determine the prevalence of migraine in a diverse community [8]. However, the prevalence is estimated to be 12–16% among women and 4–6% among men [9].

There are a number of different types of migraine, the most common being migraine without aura [10]. Migraine with aura is less common which accounts for 30–40% of cases. In this type, aura or a perceptual disturbance experienced by the patient as seeing luminous spots, feeling a particular odor, and tingling sensation in many parts of the body is absent [11]. Migraine has a significant negative impact on daily activities of the patients [12]. Unfortunately, there is no widely approved treatment for migraine and most interventions relies on headache relief or reducing the frequency and severity of attacks. The most commonly used prophylactic medications include the following: serotonin receptor agonists, beta blockers, and calcium channel blockers [13]. Drug intolerance, lack of inadequate response to drug therapy, and the high prices of medications have deprived migraineurs of a satisfactory prophylactic treatment [14]. Only 13% of all patients with a migraine sufficiently respond to conventional drug therapy [15]. Sodium valproate has been used as a prophylactic medication for migraine. Several mechanisms have been proposed for its effects, such as inhibition of gamma aminobutyric acid (GABA) transaminase and suppressing migraine-related events in the cortex, trigeminal nerve, and parasympathetic vessels. The effectiveness of sodium valproate ranged between 48 to 86.2% in various studies [16].

Statins are the first-line therapy for hypercholesterolemia and act by inhibition of β-hydroxy β-methylglutaryl-CoA (HMG-CoA) reductase [17]. While statins are best-known for their cholesterol-lowering properties, they are also thought to have pleiotropic effects. Therefore, they can improve vascular endothelial dysfunction, decrease inflammation of vascular wall and platelet aggregation, regulation of autonomic and sympathetic system and blunt thrombogenic response [18–23]. Previous studies have suggested that migrainous individuals may suffer from a generalized vasomotor and vascular dysfunction, and neuro-inflammation due to degranulation of mast cells [24–26]. Neuro-inflammation may stimulate release of vasoactive neuropeptides in trigeminal region and develop headache in migraineurs [27]. Therefore, adjuvant prophylactic therapy with known anti-inflammatory agents may help better management of migraine headache. Some clinical evidence demonstrated a direct relationship between low levels of vitamin D and headache [28]. Furthermore, vitamin D regulates production and release of pro-inflammatory cytokines and higher vitamin D concentration is associated with lower frequency of monthly migraine headache [29, 30].

Accordingly, considering the pathophysiology of migraine and the role of statins in improvement of vasomotor function, we hypothesized that they may be beneficial in prevention of migraine headache. Since atorvastatin is cost-effective and more available than other statins in our country with good penetrating properties into the CNS [31], we therefore chose this specific statin therapy for the present study. Hence, the aim of the present study was to determine whether the combination of atorvastatin and sodium valproate are beneficial in the prevention of migraine attacks in patients with migraine with aura after correction the confounding role of vitamin D deficiency. To our knowledge, this is the first a randomized, double-blind, placebo-controlled, parallel-arm study that addresses the adjuvant effects of atorvastatin in patients receiving valproate prophylactic regimen and corrected levels of vitamin D.

## Methods

The present research was a prospective, randomized triple-blind placebo-controlled trial comparing Atorvastin with placebo in prevention of migraine attacks. After obtaining approval from the Ethics Committee, Deputy of Research and Technology of Ahwaz University of Medical Sciences (Ethic code: IR. AJUMS.IREC.1396.724), the study proposal was submitted, approved, and registered by Iranian Registry of Clinical Trials (IRCT) with a registry code of IRCT20180106038242N1. This clinical trial was carried out in two medical centers of Ahwaz during 6 months (Khuzestan Province, Iran). The primary endpoint measured in this study was the number of attacks and pain severity. All patients received verbal and printed information, and all provided written consent before entry into this study. They were free to leave the study at any time during the trial if they wished.

### Inclusion criteria

The inclusion criteria were patients aged 18 to 65 years and had an established history of migraine with aura by ICHD-III criteria for at least 6 months. The patients had to have normal serum level of vitamin D3 at a serum level of ≥30 ng/mL. They were required to experience at least 3 migraine attacks monthly, but fewer than 3 high-severity-migraine attacks with negative impact on quality of life.

### Exclusion criteria

Patients were excluded from the study if they experienced chronic headaches (more than 15 attacks per month), took statins for other diseases such as diabetes, hyperlipidemia, coronary artery disease, and peripheral vascular disease, had increased liver enzymes levels of greater than twice the normal at the beginning of the study or greater than 3 times the normal during the study, were pregnant before or during the study, had presence of some degree of renal failure (estimated Glomerular Filtration Rate < 30 ml/min1.73m^2^) or creatine kinase (CK) levels of 3 times higher than the normal.

Patients also were excluded if they need to continue use of the following drugs: beta-blockers, tricyclic antidepressants, antiepileptics, calcium channel blockers, monoamine oxidase inhibitors, nonsteroidal anti-inflammatory drugs (NSAIDs), corticosteroids, or herbal remedies for migraine like St John’s wort. Beside, whenever the patients refused to sign the informed consent form or were not willing to continue the study for any reason, they were excluded from the study.

An interval of at least 1 month was given to evaluate renal and liver function, vitamin D3 and creatine kinase levels, the eligibility of patients and whether they met inclusion criteria or not. To exclude the confounding role of vitamin D deficiency on the number of migraine attacks, the vitamin D3 levels were evaluated. If the patients were vitamin D3 deficient, supplementation with 50,000 units’ soft gelatin capsule of vitamin D (ergocalciferol) was initiated and serum levels of vitamin D were corrected. The patients were also consulted for lifestyle changes (control of drug use and hormonal substances), diet (e.g. avoidance of foods which trigger migraine attacks; such as stale cheese, onion, avocado, alcoholic beverages, caffeine, and chocolate), sleep rhythm, and physical activities. After this one-month period, the patients who met all the entry criteria, were visited by the psychiatrist and neurologists (at least in a two-month period) and randomized to one of treatment groups.

### Outcome

The frequency and severity of migraine attacks were evaluated before and after administration of atorvastatin. Visual Analogue Scale (VAS) was applied to measure the severity of pain [32]. The participants were regularly monitored throughout the study for correct consumption of their medicine, possible complications, or other issues.

### Calculation of the number of patients and randomization

The sample size was calculated by using minitab software. We calculated that for a power of 0.8, significance level of 0.05, and an allowance of 10% lost to follow-up rate, for detecting a decrease in migraine attacks by one-half, 68 patients would be enough.

The eligible patients who met the criteria were assigned into one of the intervention groups, by using a permuted block randomization method. Blocks of four were used. Patients in the placebo group received sodium valproate and placebo and those in the test group were treated with sodium valproate and atorvastatin. In those patients with uncontrolled disease, if they were already taking sodium valproate, they would randomly assign into the study and continue the treatment. At the end of the study, a third party who was not involved in the study kept the randomization information confidential.

### Study design

In this prospective study, triple-blind randomization was used. Patients were assigned to receive either a combination of sodium valproate and placebo or sodium valproate and atorvastatin. Medications and placebo were quite similar in terms of shape, color, and packaging. Sodium valproate and atorvastatin tablets were purchased from Raha Pharmaceutical Co. (Isfahan, Iran) and Sobhan Darou (Tehran, Iran), respectively. Placebo tablets were prepared by a pharmaceutical specialist at Faculty of Pharmacy, Ahwaz University of Medical Sciences using starch and lactose powder (Merck, Germany) and packed in the incubation center of this university. Soft gelatin capsule of vitamin D were purchased from Dana Company (D-Vigel). Patients in the placebo group received tablets of 500-mg sodium valproate and placebo and those in the test group were treated with tablets of 500-mg sodium valproate and 20-mg atorvastatin tablets once a day for 2 months. Medication boxes were labeled “A” and “B”. Therefore, patients, treatment team, and the investigator of clinical responses were all blinded to the types of interventions. At the first visit, patients received verbal information about the study. Then, the participants were asked to fill out the VAS questionnaire in their practitioners’ presence. After 2 months, the questionnaires were filled out by the patients once again.

### Statistical analyses

Qualitative variables were reported by frequency and percentage and quantitative variables by Mean ± SD (Standard Deviation). Kolmogorov-Smirnov test and quantile-quantile plot were used to investigate the normality of data. For normally distributed data, parametric method was applied and for non-normal distribution, non-parametric approach was regarded. For univariate analysis, chi-square, fisher exact test, Pearson’s and Spearman’s rank correlation coefficient, independent sample t-test and Mann-Whitney test were used. For analysis of multiple linear regression coefficients, multiple linear, logistic, or Poisson regression were applied.

Analysis was performed on an intention-to-treat basis. All statistical analysis was conducted using SSPS software version 22 and differences with a value of *p* < 0.05 were considered to be significant.

## Results

**Fig. 1:**
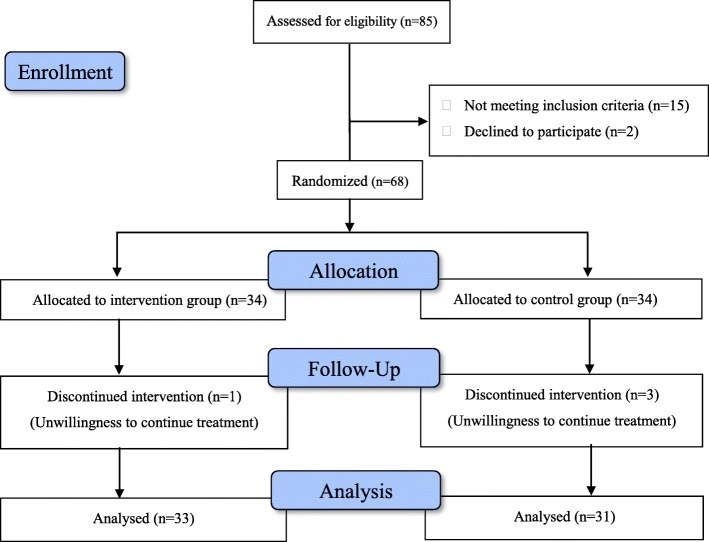
Flow diagram of the study

Based on the inclusion criteria, 68 eligible patients were randomly assigned to the two arms of the study. The flowchart of study population selection was displayed in Fig. 1. Demographic and baseline clinical characteristics of enrolled patients were presented in Table 1. All patients were followed-up regularly. However, one patient from placebo group and 3 patients from intervention group withdrew the study, because of compelling personal reasons. Compliance was good as assessed by counting medication after each follow-up visit. Both groups were similar with respect to age, gender ratio, body mass index, employment status, smoking and concomitant comorbidities (Table 1).

**Table 1:** Baseline characteristics of patients

Characteristics	Active (***n*** = 33)	Placebo (***n*** = 31)	***P***-value
**Age, median**	37.2 (23–57)	36.4 (22–52)	**0.06**
**Women, No. (%)**	22 (66.6)	21 (67.7)	**0.82**
**Employment status, No. (%)**			
Employed or student	21 (63.6)	24 (77.4)	
Household	9 (27.2)	3 (9.6)	**0.051**
Unemployed	3 (9)	4 (12.9)	
**Body mass index, median (IQR)**	18.47 (19.2–30.4)	20.67 (20.07–27.85)	**0.06**
**Smoking status, No. (%)**			
Never	20 (60)	17 (54)	
Former	5 (15)	6 (19)	**0.36**
Current	8 (24)	8 (25)	
**Comorbidities, No. (%)**			
Seasonal allergies	8 (24)	5 (16)	**0.83**
Depression	4 (12)	2 (6)	
Anxiety	5 (15)	4 (13)	
Asthma	2 (6)	3 (9)	
Emotional stress	5 (15)	6 (19)	
Sleep problems	7 (21)	8 (25)	
**Duration of migraine, No. (%)**			
≤ 2 years	19 (57.6)	17 (54.8)	**0.216**
> 2 years	14 (42.4)	14 (45.2)	
**Number of Attacks (mean ± SD)**	4.67 ± 1.05	4.61 ± 1.09	**0.743**
**Pre-treatment Median (IQR)**	5 (2)	5 (1)	
**Pain Severity (mean ± SD)**	7.85 ± 1.03	7.90 ± 0.91	**0.899**
**Pre-treatment Median (IQR)**	8 (2)	8 (2)	

*BMI* Body–mass index, *SD* Standard deviation, *IQR* Interquartile range

### The effects of atorvastatin in migraine attacks

A descriptive summary of the scores obtained on VAS questionnaire is presented in Table 2. As it is illustrated, the mean score of pain severity was 5.87 ± 1.02 in placebo group and 3.27 ± 0.88 in intervention group during 8 weeks of treatment (*p* = 0.0001). When the results were modified for other parameters, the differences between two arms of the study was yet statistically significant with the coefficient of 2.58 (*p* = 0.0001). As shown in Table 3, the preventive effects of atorvastatin on frequency of migraine attacks, in comparison to the placebo, was also statistically significant (*p* = 0.0001). The average scores of migraine attacks in atorvastatin group were 1.61 ± 0.75 over 8 weeks of the study. However, in placebo group, it was significantly higher with average scores of 3.61 ± 0.96. After controlling for possible involved variables, once more, it remained statistically significant (*p* = 0.0001). Table 4 displayed the frequency of sides effect experienced by patients. There was no remarkable difference in two arms of the study in terms of possible side effects (*P* = 0.315). 69.7% of participants in intervention group and 80.6% in the placebo group exhibited no side effect. The most common adverse effects experienced by patients were gastrointestinal symptoms, joint or skeletal pain, myalgia without CK elevation, and skin rash or itching.

**Table 2:** A summary of the scores obtained by VAS questionnaire

	Univariable		Multivariable	
	Mean ± SD Median (IQR)	***P***-value	Coefficient (95% CI)	***P***-value
**Group**				
Active	3.27 ± 0.88 3 (1)	**0.0001**	Reference	**0.0001**
Placebo	5.87 ± 1.02 6 (2)		2.58 (2.11, 3.06)	
**Sex**				
Female	4.56 ± 1.52 5 (3)	**0.771**	0.09 (−0.48, 0.65)	**0.761**
Male	4.48 ± 1.83 4 (3)		Reference	
**Marital Status**				
Single	4.67 ± 1.58 4 (3)	**0.582**	0.25 (−0.41, 0.92)	**0.452**
Married	4.45 ± 1.65 4.5 (3)		Reference	
**Duration of Migraine**				
≤2 years	4.58 ± 1.63 4.50 (3)	**0.710**	0.30 (−0.23, 0.84)	**0.262**
> 2 years	4.46 ± 1.62 4 (3)		Reference	
**Age (rho)**	- 0.05	**0.711**	0.02 (−0.02, 0.06)	**0.277**
**BMI (rho)**	- 0.06	**0.648**	- 0.03 (−0.14, 0.08)	**0.567**
**Number of attack pre-treatment (rho)**	0.15	**0.232**	0.27 (0.01, 0.54)	**0.007**

*BMI* Body–mass index, *SD* Standard deviation, *IQR* Interquartile range, *rho* Spearman correlation coefficient

**Table 3:** A summary of the scores obtained by frequency of migraine attacks

	Univariable		Multivariable	
	Mean ± SD Median (IQR)	***P***-value	Coefficient (95% CI)	***P***-value
**Group**				
Active	1.61 ± 0.75 1 (1)	**0.0001**	0.82 (0.49, 1.14)	**0.0001**
Placebo	3.61 ± 0.96 3 (1)		Reference	
**Sex**				
Female	2.44 ± 1.20 3 (2)	**0.346**	Reference	**0.706**
Male	2.86 ± 1.53 3 (3)		0.07 (−0.29, 0.43)	
**Marital Status**				
Single	2.75 ± 1.48 3 (3)	**0.502**	0.02 (−0.42, 0.45)	**0.931**
Married	2.48 ± 1.22 2.5 (2)		Reference	
**Duration of Migraine**				
≤2 years	2.64 ± 1.40 2.50 (3)	**0.765**	0.03 (−0.33, 0.39)	**0.869**
> 2 years	2.50 ± 1.23 3 (2)		Reference	
**Age (rho)**	- 0.06	**0.657**	0.003 (−0.02, 0.03)	**0.817**
**BMI (rho)**	0.02	**0.861**	0.004 (−0.07, 0.08)	**0.911**
**Number of attack pre-treatment (rho)**	0.39	**0.002**	0.21 (0.06, 0.37)	**0.007**

*BMI* Body–mass index, *SD* Standard deviation, *IQR* Interquartile range, *rho* Spearman correlation coefficient

**Table 4:** Comparison of intervention and placebo groups in terms of side effects

	Univariable				Multivariable	
	No	Yes	OR (95% CI)	***P***-value	OR (95% CI)	***P***-value
**Group**						
Active	23 (69.7%)	10 (30.3%)	1.81 (0.57,5.78)	**0.315**	1.97 (0.58,6.69)	**0.279**
Placebo	25 (80.6%)	6 (19.4%)	Reference		Reference	
**Sex**						
Female	34 (79.1%)	9 (20.9%)	Reference	**0.286**	Reference	**0.155**
Male	14 (66.7%)	7 (33.7%)	1.89 (0.59,6.07)		2.81 (0.68,11.65)	
**Marital Status**						
Single	17 (70.8%)	7 (29.2%)	1.42 (0.45,4.86)	**0.552**	0.48 (0.18,3.99)	**0.823**
Married	31 (77.5%)	9 (22.5%)	Reference		Reference	
**Duration of Migraine**						
≤2 years	26 (72.2%)	10 (27.8%)	1.41 (0.44,4.50)	**0.562**	0.94 (0.25,3.51)	**0.992**
> 2 years	22 (78.6%)	6 (21.4%)	Reference		Reference	
**Age** (mean ± SD)	37.29 ± 9.42	33.81 ± 8.89	0.96 (0.89,1.02)	**0.20**	0.97 (0.87,1.07)	**0.494**
**BMI** (mean ± SD)	24.25 ± 3.03	23.14 ± 2.99	0.88 (0.72,1.08)	**0.21**	0.88 (0.68,1.16)	**0.369**

*BMI* Body–mass index, *SD* Standard deviation, *CI* Confidence interval

The patient satisfaction was compared between the two groups using Fisher’s exact test. As shown in Table 5, there was a significant difference between two groups of the study in terms of patient satisfaction; as 90.9% of participants in atorvastatin group and only 51.6% in placebo group were satisfied with their medications (*p* = 0.001). After controlling for age, sex, body mass index (BMI), marital status and duration of migraine, the difference between two groups were yet significant with the odds ratio of 9.83 (*p* = 0.001).

**Table 5:** Comparison of test and placebo groups in terms of patient satisfaction

	Univariable				Multivariable	
	No	Yes	OR (95% CI)	***P***-value	OR (95% CI)	***P***-value
**Group**						
Active	3 (9.1%)	30 (90.9%)	9.38 (2.36,37.3)	**0.001**	9.83 (2.40,40.27)	**0.001**
Placebo	15 (48.4%)	16 (51.6%)	Reference		Reference	
**Sex**						
Female	11 (25.6%)	32 (74.4%)	1.46 (0.47,4.53)	**0.518**	1.77 (0.43,7.33)	**0.430**
Male	7 (33.3%)	14 (66.7%)	Reference		Reference	
**Marital Status**						
Single	7 (29.2%)	17 (70.8%)	Reference	**0.886**	Reference	**0.547**
Married	11 (27.5%)	29 (72.5%)	1.09 (0.35,3.33)		1.75 (0.28,10.88)	
**Duration of Migraine**						
≤2 years	10 (27.8%)	26 (72.2%)	1.04 (0.35,3.12)	**0.944**	1.10 (0.27,4.46)	**0.891**
> 2 years	8 (28.6%)	20 (71.4%)	Reference		Reference	
**Age** (mean ± SD)	37.39 ± 9.17	36.04 ± 9.48	0.98 (0.93,1.04)	**0.602**	0.96 (0.86,1.06)	**0.399**
**BMI** (mean ± SD)	24.06 ± 2.85	23.94 ± 3.14	0.99 (0.82,1.18)	**0.987**	1.04 (0.77,1.40)	**0.811**

*BMI* Body–mass index, *SD* Standard deviation, *CI* Confidence interval

## Discussion

Within the 2 months of intervention, atorvastatin at dose of 20 mg/day showed a significant reduction in the number of migraine attacks in migraine patients with aura. Furthermore, treatment with atorvastatin at this dose was associated with significant improvement in pain severity during the 8 weeks of clinical trial. Most adverse effects experienced by the patients in both arms were mild and tolerable and no specific side effect was observed during the study. The number of patients experiencing adverse effects were slightly higher in intervention group, although this difference did not reach a statistical significance.

Addition of atorvastatin in preventive regimen of migraineurs with aura was associated with a responder rate of 65% during the 2 months of study and a mean reduction of 3 migraine attacks per month. These amounts in previous studies were 50% for propranolol [33], amitriptyline [34], sodium valproate and divalproex and 40% for candesartan [35]. Patient satisfaction, as an indicator of quality care, was significantly higher in intervention group. This could be attributed to the reduced number of migraine attacks following the addition of atorvastatin to the treatment.

Some studies have shown that migraine patients may suffer from endothelial dysfunction of cerebral, coronary, retinal, dermal and peripheral vasculature [36]. They believe that the origin of migraine is neurologic inflammation in central nervous system. Migraineurs may have impaired endothelium dependent function and an underlying systemic vasomotor abnormality [37]. They have a diminished endothelium-dependent vasodilatation capacity compared with healthy-participants. Therefore, statins with their proven improving effects on endothelial and vasomotor function, attenuating oxidative stress and inflammatory cytokines in central brain, and neuronal protection could be beneficial in prevention of migraine headache [38]. Atorvastatin, the most commonly used drug among statins, suppresses nuclear factor κB pathway in trigeminal nucleus, which has a critical role in pathogenesis of migraine in recent studies [39–41].

A recent study has shown that atorvastatin is as effective and safe as sodium valproate in preventing migraine attacks. They believed that this may contributed to antinociceptive, anti-inflammatory and antioxidant effects of statins. Moreover, administration of this medication was not accompanied by any specific adverse effects in patients [42]. In a case report, it was indicated that initiation of atorvastatin at dose of 20 mg/day for a patient with frequent attacks of typical migraine completely resolved migraine attacks [43]. In another study, it was reported that twice daily consumption of 1000 international units’ vitamin D3 and 20 mg of simvastatin for 12 weeks significantly reduced the number of migraine attacks in patients with more than 10 years history of migraine [44]. However, their study design could not differentiate between an effect of statin alone, or vitamin D alone, or the combination of both of them. However, in the present study, an interval of at least 1 month was set to correct and obtain a normal serum level of vitamin D3 before allocation into study arms in those patients with vitamin D deficiency. Therefore, the possible effect of vitamin D was diminished.

In a study by Pahan et al., it was shown that statins induce iNOS expression, upregulate endothelium nitric oxide synthase, which result in increased nitric oxide levels [45]. This may offset the diminished vasodilatation capacity of patients and explain the decreased number of migraine attacks and their severity observed in the intervention arm. It seems that atorvastatin prevents migraine attacks by improving the vasomotor performance in cerebral vessels [46, 47].

One of the major limitation of the present study was not therapeutically monitoring the serum levels of valproic acid. Due to the remarkable inter-individual pharmacokinetic variability of valproic acid, it is important to follow the patients for the efficacy of treatment and possible toxicity. However, monitoring for valproic acid concentration was not available in most laboratories of the city. Therefore, we enrolled only patients with normal renal and liver function who are at lower risk of experiencing side effects with pharmacotherapy. The patients were also orally informed about possible side effects and they were questioned on follow-up visits for presenting any side effects. The second limitation of the study was the small number of patients, since some serious side effects reported with statins, e.g. CK elevation, are very rare. Thereby, the impact of such events on patients’ final outcome needs to be specified. Third, the number of adverse effects experienced by patients was very small and they could not be analyzed separately. Therefore, we analyzed all the observed side effects in general and together. Although the results of the present study were so promising with predefined endpoints and controlled triple blinded trial manner, these findings should be confirmed in larger groups of migraineurs with and without aura in longer duration of follow-up to optimize the best dose of atorvastatin and optimal period of treatment for better prevention of migraine headache and migraine attacks. In future studies, we also suggest to assess the impact of pleiotropic effects of statins for prevention of migraine in appropriate models.

## Conclusion

In summary, the data of this study support that atorvastatin at dose of 20 mg/day could be considered as a reasonable therapeutic option for prophylactic treatment of migraine headache with aura. This medication was safe and effective and well tolerated by migraineurs and significantly reduced the number and severity of migraine attacks, without causing any significant adverse events.

## Data Availability

The datasets used and/or analysed during the current study available from the corresponding author on reasonable request. The raw SPSS file of this study before analysis is available upon your request.

## Abbreviations

GABA: Gamma aminobutyric acid
HMG-CoA: β-hydroxy β-methylglutaryl-CoA
NSAIDs: Nonsteroidal anti-inflammatory drugs
VAS: Visual Analogue Scale
BMI: Body mass index
